# Direct Z-Scheme M_2_X/BiOY (M = Ag, Au; X = S, Se; Y = Cl, Br, I) Heterojunctions for Solar-Driven Photocatalytic Water Splitting Applications: A First-Principles Investigation

**DOI:** 10.3390/nano15110844

**Published:** 2025-06-01

**Authors:** Qiyun Deng, Lei Gao, Wuyi Gao, Jiali Hao, Chunhua Zeng, Hua Wang

**Affiliations:** 1 Institute of Physical and Engineering Science, Faculty of Science, Kunming University of Science and Technology, Kunming 650500, China; dengqiyun@stu.kust.edu.cn (Q.D.); gaowuyi@stu.kust.edu.cn (W.G.); haojiali@stu.kust.edu.cn (J.H.); chzeng83@kust.edu.cn (C.Z.); 2 State Key Laboratory of Complex Nonferrous Metal Resources Clean Utilization, Kunming University of Science and Technology, Kunming 650093, China

**Keywords:** heterojunction photocatalyst, solar-driven water splitting, direct Z-scheme, first-principles calculations

## Abstract

Two-dimensional direct Z-scheme photocatalysts have emerged as highly promising photocatalysts for solar-driven water splitting owing to their effective separation of photogenerated carriers and strong redox abilities. This study focuses on the theoretical prediction of promising Z-scheme photocatalysts for solar-driven water splitting based on M_2_X/BiOY (M = Ag, Au; X = S, Se; Y = Cl, Br, I) heterojunctions using first-principles calculations. All M_2_X/BiOY heterojunctions possess staggered band alignments, Z-scheme carrier migration, and suitable band edges for overall water splitting. Optical absorption spectra indicate that these heterojunctions exhibit significantly extended solar absorption in the visible and near-infrared regions. Moreover, the interfacial built-in electric fields of (0.46–0.72 V/Å) point from M_2_X to BiOY, promote photogenerated carrier separation, and enhance redox overpotentials, thereby improving photocatalytic performance. These results suggest that M_2_X/BiOY heterojunctions are promising Z-scheme photocatalysts for solar-driven water splitting and are expected to be experimentally prepared and realized in the near future.

## 1. Introduction

Solar-driven photocatalytic water splitting for hydrogen production is a sustainable way to meet critical energy needs without harmful emissions [1,2,3]. Two-dimensional semiconductors have been widely investigated as photocatalysts for solar-driven water splitting due to their large specific surface areas, abundant active sites, and short carrier migration distances [4,5,6]. However, most monolayer photocatalysts face severe problems with photogenerated electron–hole recombination and mutual contradiction between broad optical absorption and strong redox capability [7,8,9,10,11,12,13]. To address these limitations, inspired by the natural photosynthesis of green plants, researchers proposed a direct Z-scheme photocatalytic system to overcome these drawbacks [14,15,16,17,18,19,20,21]. In the direct Z-scheme photocatalysts, electrons and holes are spatially separated with higher redox potentials, while solar absorptions extend significantly into the visible and near-infrared regions, ultimately enhancing the photocatalytic performance [22,23,24,25,26,27,28]. Therefore, constructing direct Z-scheme heterojunctions is a feasible strategy to improve the solar-driven photocatalytic water-splitting performance significantly.

Recently, 2D semiconducting group-11 chalcogenides (M_2_X; M = Cu, Ag, Au; X = S, Se, Te) with high carrier mobilities and wide bandgap ranges have been experimentally and theoretically reported [29,30,31,32,33,34]. Their band edges are suitable for half or overall redox reaction, indicating that they are promising to form direct Z-scheme photocatalysts for high-performance photocatalytic water splitting. Meanwhile, 2D bismuth oxyhalides (BiOY; Y = Cl, Br, I) are also widely investigated photocatalysts for water splitting [35,36,37,38,39]. Inspired by the potential applications of 2D group-11 chalcogenides and bismuth oxyhalides, further investigations in M_2_X/BiOY (M = Ag, Au; X = S, Se; Y = Cl, Br, I) heterojunctions via first-principles calculations are highly desired to provide references for experimental researchers to search for efficient solar-driven water-splitting photocatalysts more effectively.

In this study, we systematically investigate the photocatalytic properties of M_2_X/BiOY (M = Ag, Au; X = S, Se; Y = Cl, Br, I) heterojunctions via first-principles calculations. The results demonstrate that all M_2_X/BiOY heterojunctions exhibit staggered band alignments, which are favorable for the spatial separation of photogenerated carriers. An intrinsic electric field, *E_in_* is present at the interfaces, directed from M_2_X towards BiOY, with field strengths ranging from 0.46 V/Å to 0.72 V/Å. On the driving force of the *E_in_*, the carrier migration mechanisms of M_2_X/BiOY heterojunctions are all direct Z-schemes, which are beneficial for achieving high performance for overall water splitting owing to the effective separation of photogenerated carriers and the strong redox abilities. Furthermore, the optical absorbances of M_2_X/BiOY heterojunctions are enhanced in comparison to those of their isolated monolayers. And the first absorption peaks of Au_2_Se/BiOCl, Au_2_Se/BiOBr, and Au_2_Se/BiOI are in the infrared range of 1.31 eV, 1.40 eV, and 1.48 eV, respectively. The enhanced optical absorbances of M_2_X/BiOY heterojunctions further resulted in their better photocatalytic performances.

## 2. Materials and Methods

All calculations in this paper were based on the density functional theory (DFT) method in the Vienna ab initio simulation package (VASP) program package [40,41,42,43], version 5.4.4. The exchange-correlation potential was employed by the Perdew–Burke–Ernzerhof (PBE) of the Generalized Gradient Approximation (GGA) [44]. The cut-off energy for the plane-wave basis set was 600 eV. The conjugate gradient scheme was used for geometry optimizations, and the convergence criteria for the energy and ionic Hellmann-Feynman force were set to 10^−5^ eV/atom and 0.01 eV/Å, respectively. In all calculations, a vacuum layer of at least 15 Å along the z-direction was used to eliminate interlayer interactions. As a better description of the weak van der Waals forces between layered 2D materials, the DFT-D3 method with Becke–Johnson damping has been adopted [45,46,47]. The first Brillouin zone was sampled using a Γ-centered 6 × 6 × 1 grid for the geometrical optimization and calculations of the electronic properties. In addition, the Heyd–Scuseria–Ernzerhof (HSE06) [48] was adopted to compute the electronic and optical properties due to the fact that the PBE functional will underestimate the bandgap in semiconductors.

## 3. Results

**Figure 1:**
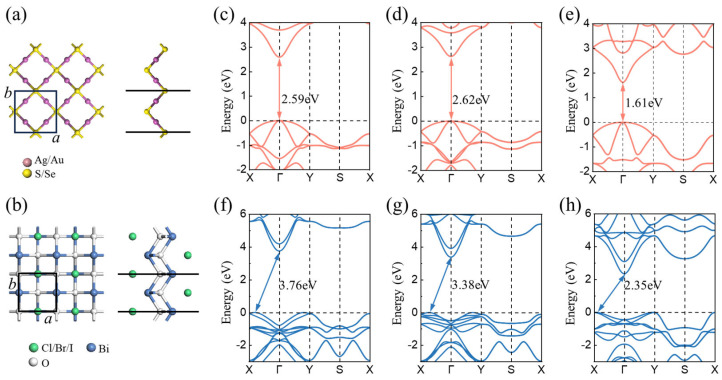
Top and side views of monolayer (**a**) M_2_X (M = Ag, Au; X = S, Se) and (**b**) BiOY (Y = Cl, Br, I), respectively. The black squares represent the unit cells. Band structures of monolayer (**c**) Ag_2_S, (**d**) Ag_2_Se, (**e**) Au_2_Se, (**f**) BiOCl, (**g**) BiOBr, and (**h**) BiOI, respectively.

The atomic and electronic structures of monolayer M_2_X (M = Ag, Au; X = S, Se) and BiOY (Y = Cl, Br, I) were first investigated, as illustrated in Figure 1. The unit cells of M_2_X and BiOY are both squares with the same space group of P4/nmm. The optimized lattice constants of Ag_2_S, Ag_2_Se, Au_2_Se, BiOCl, BiOBr, and BiOI are 5.84, 5.90, 5.82, 3.96, 3.98, and 4.03 Å, respectively. However, the PBE method usually overestimates the lattice constant [49,50,51], resulting in a deviation from the experimentally reported values. The band structures of each monolayer were calculated based on the HSE06 hybridization method. The results demonstrate that Ag_2_S, Ag_2_Se, and Au_2_Se are all direct bandgap semiconductors with valence band maximums (VBMs) and conduction band minimums (CBMs) at the Γ point. Their bandgaps are 2.59, 2.62, and 1.61 eV, respectively. BiOCl, BiOBr, and BiOI are indirect bandgap semiconductors with bandgaps of 3.76, 3.38, and 2.35 eV, respectively. Compared with previously reported bulk BiOX [52], the calculated band gap values in this study show certain differences due to quantum confinement effects [53], as illustrated in Table S1. Their VBMs and CBMs are located at the X-Γ line and Γ point, respectively.

**Figure 2:**
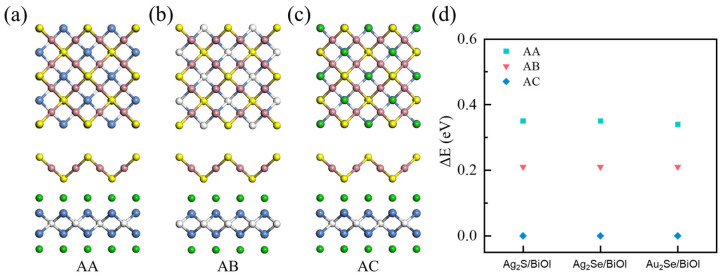
Top and side views of Ag_2_S/BiOI heterojunctions with (**a**) AA, (**b**) AB, and (**c**) AC stackings, respectively. (**d**) Relative energies of AA, AB, and AC stackings for Ag_2_S/BiOI, Ag_2_Se/BiOI, and Au_2_Se/BiOI heterojunctions.

Due to the large differences in lattice constants between M_2_X and BiOY, M_2_X/BiOY heterojunctions were constructed by stacking 1 × 1 M_2_X on √2 × √2 BiOY to achieve a better lattice match, since strain induced by lattice mismatch will affect the stabilities and charge transfer dynamics of M_2_X/BiOY heterojunctions. Considering the stacking pattern has a tremendous influence on the stabilities and charge transfer dynamics of M_2_X/BiOY heterojunctions, the total energies, charge density differences, and projected band structures of Ag_2_S/BiOI with different stacking patterns (AA, AB, and AC) are compared in Figure 2, Figures S1 and S2. All Ag_2_S/BiOI with different stacking patterns are staggered band alignments. The charge density difference of the energetically favorable AC stacking is stronger than those of AA and AB stackings, indicating that the energetically favorable AC stacking is the most conducive to interlayer charge transfer in Ag_2_S/BiOI. Additionally, the lowest energies for Ag_2_Se/BiOI and Au_2_Se/BiOI are also observed in AC stacking. In the following, the AC stacking is used for all M_2_X/BiOY heterojunctions to further explore their structural, electronic, optical, and photocatalytic properties.

The structural stabilities of M_2_X/BiOY heterojunctions are judged by the binding energy ($E_{b}$), whose calculation formula is as follows:

(1) $$E_{b}=E_{heter}-E_{A}-E_{B}$$

where $E_{heter}$, $E_{A}$, and $E_{B}$ are the total energies of the heterostructures, isolated M_2_X, and BiOY monolayers, respectively. The E_b_ of M_2_X/BiOY heterojunctions ranges from −2.592 to −3.646 eV (see Table S2), whose negative values indicate these stable contacts are easy to form. Meanwhile, taking Ag_2_S/BiOI as an example, its thermodynamic stability is further evaluated by AIMD simulation and phonon dispersion calculation, as shown in Figure S3. A 3 × 3 × 1 supercell with 162 atoms is built to ensure the accuracy of the simulation. After 2 ps simulation at 300 K, the structure of Ag_2_S/BiOI is not significantly damaged. The integrity of the original structure during time evolution confirms its excellent thermal stability. Furthermore, the absence of any significant imaginary phonon modes indicates its dynamic stability. Since the non-layer bulk phases of M_2_X, it is suggested that they can be synthesized with molecular beam epitaxy (MBE) or chemical vapor deposition (CVD) methods, which have been extensively utilized for exploration of new 2D materials and heterostructures [54,55]. Considering the rapid development of experimental techniques for fabricating 2D materials and heterostructures in recent years, we are optimistic that these M_2_X/BiOY heterojunctions can be fabricated experimentally in the near future.

**Figure 3:**
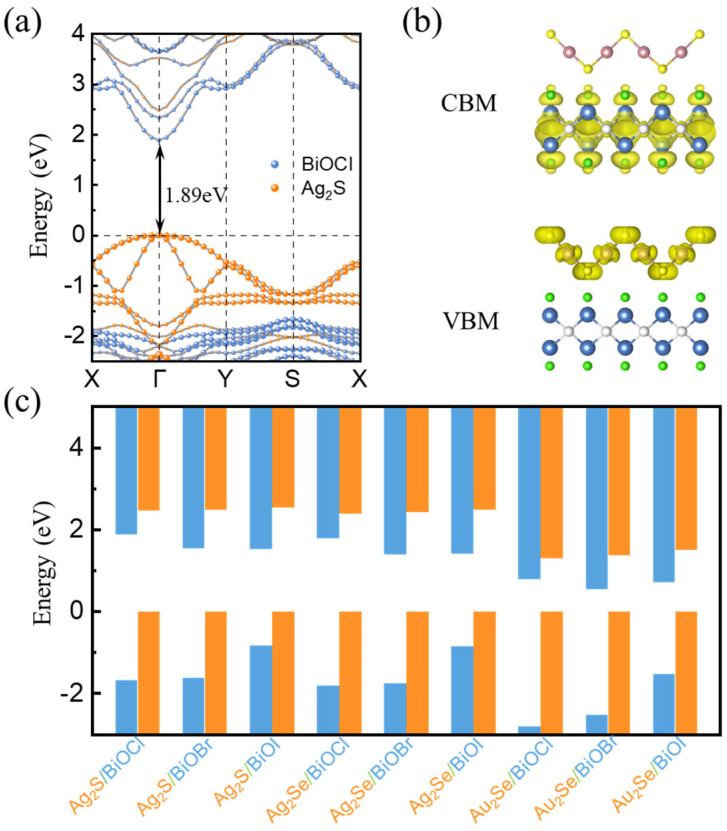
(**a**) Projected band structure and (**b**) partial charge density of the Ag_2_S/BiOCl heterojunction, respectively. The yellow region represents the spatial distribution of the electronic states corresponding to the conduction band minimum (CBM) and valence band maximum (VBM). (**c**) Band edges of M_2_X/BiOY heterojunctions with VBMs set to 0.

The electronic structures and band alignments of heterojunctions are important for their applications. In order to have a comprehensive understanding of the electronic properties of M_2_X/BiOY heterojunctions, we calculated the projected band structures of each heterojunction based on the HSE06 method, as shown in Figure 3a and Figure S4. All heterojunctions possess direct bandgaps with the CBMs and VBMs located at the Γ point. We also calculated the partial charge densities of these heterojunctions, as shown in Figure 3b and Figure S5. The CBMs and VBMs of M_2_X/BiOY heterojunctions are mainly contributed by BiOY and M_2_X, respectively, which suggests that M_2_X/BiOY heterojunctions are staggered band alignments. In these heterojunctions, electrons and holes are separated into different layers. Band edges of M_2_X/BiOY heterojunctions with VBMs set to 0 are summarized in Figure 3c. These M_2_X/BiOY heterojunctions are all staggered band alignments, which is beneficial to hinder photogenerated carrier recombination.

**Figure 4:**
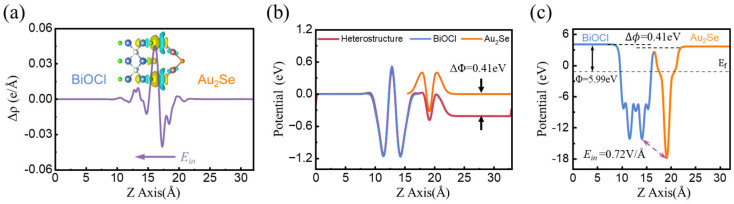
Plane-averaged (**a**) charge density difference, (**b**) Hartree difference potential, and (**c**) electrostatic potential of Au_2_Se/BiOCl heterojunction, respectively.

When M_2_X and BiOY come into contact, internal built-in electric fields inevitably form. It has been well established that the interface electric field in staggered-band-alignment heterojunction plays a crucial role in facilitating the separation of photogenerated charge carriers in photocatalysts [56,57]. This interfacial electric field arises from charge redistribution at the interface, resulting from interlayer interactions. To investigate interfacial electric fields at the M_2_X/BiOY interface, the charge density difference along the z direction was calculated using the following formula:

(2) $$\Delta \rho =\rho_{heterojunction}-\rho_{monolayer1}-\rho_{monolayer2}$$

where $\rho_{heterojunction}$, $\rho_{monolayer1}$, and $\rho_{monolayer2}$ represent the charge density of M_2_X/BiOY, isolated monolayer M_2_X, and BiOY, respectively. The planar average charge density difference of M_2_X/BiOY heterojunctions is presented in Figure 4a and Figure S6. The corresponding three-dimensional schematics of the charge density difference depict charge accumulation and depletion regions, marked in yellow and cyan, respectively. Notably, these results demonstrate significant charge transfer between the layers of the heterojunction. Taking Au_2_Se/BiOCl as an example, the electrons accumulate in BiOCl and are depleted in Au_2_Se. This charge redistribution results in the formation of an interlayer electric field directed from Au_2_Se to BiOCl. Similarly, other heterojunctions exhibit the same charge transfer behavior, indicating the presence of an electric field across the interfaces, directed from M_2_X to BiOY. Furthermore, we provide the Hartree potential difference to further confirm the direction of the electric field, as shown in Figure 4b and Figure S7. Here, ∆Φ represents the effect of the internal electric field, defined as the difference between the sum of the ∆φ values of isolated monolayers and that of the heterojunction. The ∆Φ of Au_2_Se/BiOCl is 0.41 eV, so the internal electric field is directed from Au_2_Se to BiOCl, in agreement with the charge density difference results.

Furthermore, the interfacial charge transfer can be further investigated using the work function Φ, which is defined as follows:

(3) $$\Phi =E_{vac}-E_{F}$$

where $E_{vac}$ and $E_{F}$ represent the vacuum and Fermi energy levels, respectively. The work function values of Ag_2_S, Ag_2_Se, Au_2_Se, BiOCl, BiOBr, and BiOI are 5.97, 5.87, 5.02, 7.78, 7.57, and 6.54 eV, respectively. As shown in Figure S8, when M_2_X and BiOY form M_2_X/BiOY heterojunctions, electrons transfer from M_2_X (with the lower work function) to BiOY (with the higher work function) in order to align their Fermi levels, ultimately resulting in the formation of an internal electric field (*E_in_*) at the interface, directed from M_2_X to BiOY. The plane-averaged electrostatic potential of the Au_2_Se/BiOCl heterojunction, as well as other systems, is shown in Figure 4c and Figure S9. Due to charge transfer, a difference of ∆*Φ* = 0.41 eV forms across the Au_2_Se/BiOCl heterojunction upon contact between the materials, corresponding to the ∆*Φ* value presented in Figure 4b. Meanwhile, a potential difference exists between Au_2_Se and BiOCl, corresponding to an intrinsic electric field *E_in_* of 0.72 V/Å in quantitative terms.

**Figure 5:**
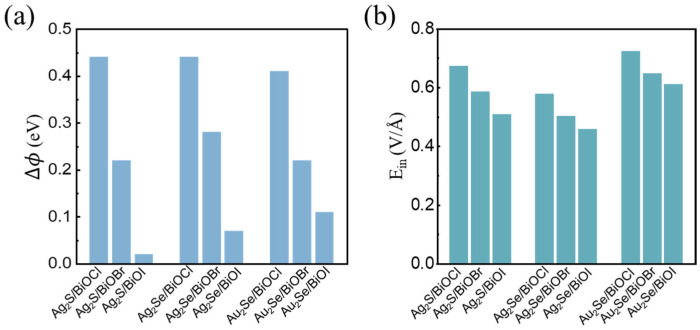
(**a**) Potential differences (∆*Φ*) and (**b**) interfacial built-in electric fields (*E_in_*) of M_2_X/BiOY heterojunctions.

Figure 5 summarizes the ∆*Φ* and *E_in_* values for M_2_X/BiOY heterojunctions. The ∆*Φ* values of these heterojunctions range from 0.02 eV to 0.44 eV. As the radius of the Y atom increases, the ∆*Φ* of M_2_X/BiOY heterojunctions decreases. Notably, the ∆*Φ* values of Ag_2_S/BiOCl, Ag_2_Se/BiOCl, and Au_2_Se/BiOCl are significantly larger than those of the other systems. The *E_in_* values of the M_2_X/BiOY heterojunctions range from 0.46 V/Å to 0.72 V/Å, which are comparable to those reported in 0.832 V/Å [58], 0.62 V/Å [59], and 0.422 V/Å [60]. A large *E_in_* can facilitate the separation of photogenerated electron–hole pairs, thereby enhancing photocatalytic efficiency.

**Figure 6:**
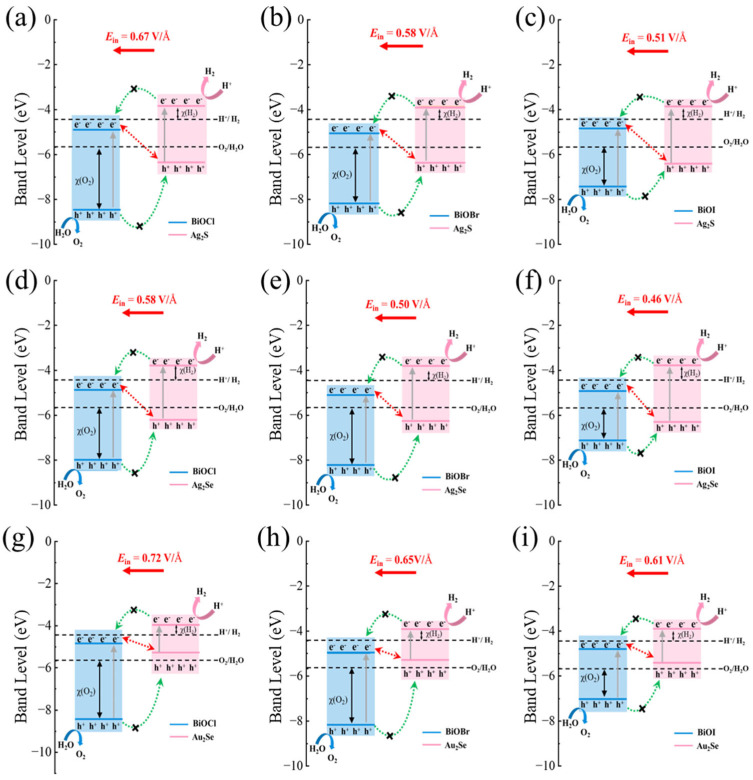
Band edge positions and carrier migration mechanisms of (**a**) Ag_2_S/BiOCl, (**b**) Ag_2_S/BiOBr, (**c**) Ag_2_S/BiOI, (**d**) Ag_2_Se/BiOCl, (**e**) Ag_2_Se/BiOBr, (**f**) Ag_2_Se/BiOI, (**g**) Au_2_Se/BiOCl, (**h**) Au_2_Se/BiOBr, and (**i**) Au_2_Se/BiOI, respectively. The direct Z-scheme and type-II carrier migration pathways are presented by red dashed lines with double arrows and green dashed lines with single arrows, respectively. The χ(H_2_) and χ(O_2_) indicate the overpotential of the reduction and oxidation reactions at pH = 0, respectively.

Based on the *E_in_* and band edge positions of M_2_X/BiOY heterojunctions, carrier migration pathways at their interfaces are further analyzed, as shown in Figure 6. All band edge positions and redox potentials are plotted relative to the vacuum level, with an aligned Fermi level for each heterojunction. Taking Ag_2_S/BiOCl as an example, the feasibility of photocatalytic water splitting was determined by comparing the CBM and VBM of each component material of the heterojunction with the redox potential of water splitting. Specifically, if electrons and holes migrate to the CBM of BiOCl and the VBM of Ag_2_S, respectively, the charges satisfy the type-II migration path (shown by the green dashed single arrows in Figure 6a). Here, the CBM of the system is below the reduction potential of water and cannot satisfy the potential requirement for the HER reaction. In contrast, if the electrons are retained in the CBM of Ag_2_S and the holes are located in the VBM of BiOCl, the band edge positions can satisfy the potential requirements for both HER and OER reactions, which is consistent with the direct Z-scheme charge transfer mechanism. Due to the driving force of the *E_in_*, which points from Ag_2_S to BiOCl, photogenerated electrons at the CBM of BiOCl will combine with photogenerated holes at the VBM of Ag_2_S, which promotes the formation of a direct Z-scheme carrier migration path. Simultaneously, the *E_in_* also restrains the migration of photogenerated electrons from the CBM of Ag_2_S to the CBM of BiOCl and the migration of photogenerated holes from the VBM of BiOCl to the VBM of Ag_2_S, thereby hindering carrier transfer along the type-II pathway. Owing to possessing a carrier migration mechanism of direct Z-scheme, HER and OER occur on the CBM of Ag_2_S and the VBM of BiOCl, respectively, where the higher CBM and the lower VBM result in larger overpotentials for HER χ(H_2_) and OER χ(O_2_), respectively. These increased overpotentials reflect stronger redox driving forces of the photogenerated carriers, thereby significantly enhancing the photocatalytic performance. Similar redox behavior is observed in other M_2_X/BiOY heterojunctions. Similarly, all M_2_X/BiOY heterojunctions are promising direct Z-scheme photocatalysts, with overpotentials for the HER χ(H_2_) and OER χ(O_2_) ranging from 0.48 to 0.65 eV and from 1.34 to 2.79 eV, respectively. Therefore, Z(S)-scheme M_2_X/BiOY heterojunctions are beneficial for achieving high performance for overall water splitting owing to the effective separation of photogenerated carriers and the strong redox abilities.

**Figure 7:**
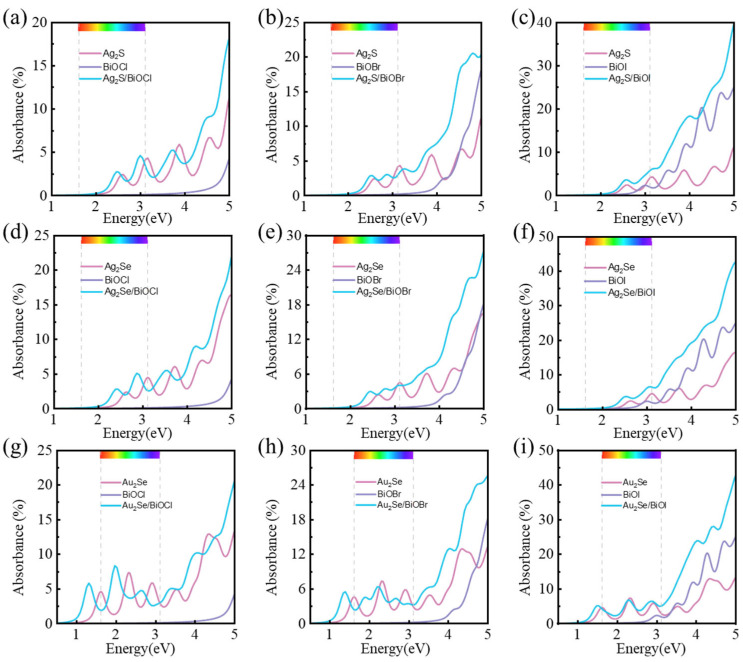
Optical absorbances of (**a**) Ag_2_S/BiOCl, (**b**) Ag_2_S/BiOBr, (**c**) Ag_2_S/BiOI, (**d**) Ag_2_Se/BiOCl, (**e**) Ag_2_Se/BiOBr, (**f**) Ag_2_Se/BiOI, (**g**) Au_2_Se/BiOCl, (**h**) Au_2_Se/BiOBr, and (**i**) Au_2_Se/BiOI, respectively.

The optical properties of photocatalysts are crucial for the performance of photocatalytic water splitting, as they determine the materials’ ability to effectively absorb and utilize visible light, which directly influences the efficiency of the photocatalytic reaction. Consequently, we calculated the optical absorbance of the M_2_X/BiOY heterojunction using the following equation:

(4) $$A\left(\omega \right)=\varepsilon_{2}\left(\omega \right)L_{z}\frac{\omega }{c}$$

where $L_{z}$, *ω*, and *c* represent the unit cell thickness along the *z*-axis, the phonon frequency, and the speed of light, respectively. As shown in Figure 7. The optical absorbances of M_2_X/BiOY heterojunctions are enhanced in a wide range of visible and near-ultraviolet light, in comparison to those of their isolated monolayers. And the absorption edges of all heterojunctions show redshift. Notably, the first absorption peaks of Au_2_Se/BiOCl, Au_2_Se/BiOBr, and Au_2_Se/BiOI are observed in the near-infrared range, at 1.31 eV, 1.40 eV, and 1.48 eV, respectively. Besides the visible and ultraviolet regions, these heterojunctions also exhibit solar absorption in the near-infrared region. The enhanced optical absorbances of M_2_X/BiOY heterojunctions further resulted in their better photocatalytic performances.

**Figure 8:**
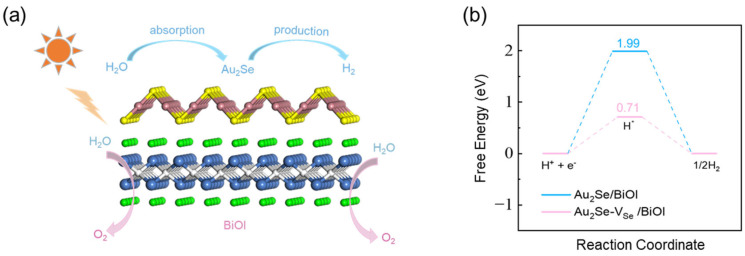
(**a**) Schematic diagram of the photocatalytic hydrogen production mechanism in the heterojunction. (**b**) Gibbs free energy changes $∆G_{H^{*}}$ of HER processes. Pink and cyan lines represent $∆G_{H^{*}}$ of Au_2_Se surface with and without Se vacancy, respectively, at pH = 0.

Furthermore, the activities of HER and OER reactions in Au_2_Se/BiOI are investigated. Through previous analysis, we know that the HER reaction occurs in Au_2_Se and the OER reaction occurs in BiOI, as shown in Figure 8a. Since 2D BiOX (X = Cl, Br, I) has been widely reported in the field of photocatalytic oxygen evolution [61,62,63,64,65,66], here we focus on the HER on the Au_2_Se side of Au_2_Se/BiOI, which is estimated by Gibbs free energies of the intermediate reactants (Figure 8b). Au_2_Se/BiOI exhibits a high $∆G_{H^{*}}$ value of 1.99 eV at pH = 0, which results in suppression of HER. To enhance catalytic efficiency, the modulation of single-atom vacancy is an effective method. We introduce the Se vacancy into Au_2_Se/BiOI via removing one Se atom from the Au_2_Se surface in a 2 × 2 supercell. The value of $∆G_{H^{*}}$ decreases significantly to 0.71 eV, indicating that Se atomic vacancy can substantially enhance its catalytic performance. These results suggest that Au_2_Se/BiOI is a promising photocatalyst for overall water splitting.

## 4. Conclusions

In this study, we design a novel M_2_X/BiOY(M = Ag, Au; X = S, Se; and Y = Cl, Br, I) heterojunction and investigate its potential as an efficient Z-scheme photocatalyst for solar-driven overall water splitting. Using first-principles calculations, we systematically investigated the electronic structure, optical properties, and photocatalytic performance of these heterojunctions. The results show that all heterojunctions are staggered-band-alignment heterojunctions, which are favorable for the spatial separation of photogenerated carriers. Furthermore, we identified the presence of an internal electric field *E_in_* at the heterojunction interface, pointing from M_2_X to BiOY, with electric field strengths between 0.46 V/Å and 0.72 V/Å. This electric field promotes the recombination of charge carriers with low oxidation–reduction capabilities while retaining the charge carriers with strong redox abilities. This internal field facilitates the recombination of charge carriers with lower redox potential while preserving those with higher redox potential. Consequently, the hydrogen evolution reaction (HER) occurs at the conduction band minimum (CBM) of M_2_X, while the oxygen evolution reaction (OER) takes place at the valence band maximum (VBM) of BiOY. This Z-scheme mechanism increases the overpotentials for both HER χ (H_2_) and OER χ (O_2_), enhancing the overall photocatalytic efficiency. Furthermore, compared to their respective isolated monolayers, M_2_X/BiOY heterojunctions exhibit significantly stronger optical absorption in the visible and near-ultraviolet light regions. Specifically, the first absorption peaks for Au_2_Se/BiOCl, Au_2_Se/BiOBr, and Au_2_Se/BiOI heterojunctions are located at 1.31 eV, 1.40 eV, and 1.48 eV, respectively, in the infrared region. These enhanced optical absorption properties further promote their photocatalytic performance. Finally, the Gibbs free energy of the hydrogen evolution reaction for the Au_2_Se/BiOI heterojunction. The results further indicate that M_2_X/BiOY heterojunctions hold significant promise as novel Z-scheme photocatalysts for water splitting applications.

## Data Availability

Data are contained within the article or Supplementary Materials. The data that support the findings of this study, including input files for the first-principles calculations, are available from the corresponding author upon reasonable request.

## Supplementary Materials

The following supporting information can be downloaded at: https://www.mdpi.com/article/10.3390/nano15110844/s1. Figure S1: Projected band structures of Ag_2_S/BiOI with (a) AA, (b) AB and (c) AC stackings, respectively; Figure S2: Charge density differences ∆ρ of Ag_2_S/BiOI with (a) AA, (b) AB and (c) AC stackings, respectively; Figure S3: (a) Top and side views of the snapshots of Ag_2_S/BiOI taken from ab initio molecular dynamic simulations carried out at 300 K for 2 ps. (b) Phonon dispersions of Ag_2_S/BiOI; Figure S4: Projected band structures of M_2_X/BiOY heterojunctions. The orange and blue colors represent the M_2_X monolayer and the BiOY monolayer, respectively; Figure S5: Partial charge densities of M_2_X/BiOY heterojunctions; Figure S6: Planar-averaged charge density differences along the z direction of M_2_X/BiOY hetero-junctions; Figure S7. Plane-averaged Hartree difference potentials along the z direction of M_2_X/BiOY heterojunctions; Figure S8: Fermi level change in M_2_X/BiOY before contact and after contact; Figure S9. Plane-averaged electrostatic potential along the z direction of M_2_X/BiOY heterojunctions. E_f_ represents the Fermi level and the black arrow indicates the difference between the Fermi level and the vacuum level. The vacuum energy level difference between the two sides of the heterojunction is represented by ∆*Φ*. Table S1: Comparison of Band Gaps between bulk and monolayer BiOX; Table S2: The binding energy (E_b_) of M_2_X/BIOY heterojunctions.
