# Supplementary material for: Direct Z-Scheme M2X/BiOY (M = Ag, Au; X = S, Se; Y = Cl, Br, I) Heterojunctions for Solar-Driven Photocatalytic Water Splitting Applications: A First-Principles Investigation
Source: Nanomaterials (Basel). 2025 Jun 1;15(11):844. doi: 10.3390/nano15110844 (PMC12157931; doi:10.3390/nano15110844)
Supplement: Supplementary file 1 [file nanomaterials-15-00844-s001.zip › nanomaterials-3608065-supplementary.pdf]

nanomaterials

Supplementary Information

Direct Z-Scheme M_2X/BiOY (M = Ag, Au; X = S, Se; Y = Cl, Br, I) Heterojunctions for Solar-Driven Photocatalytic Water Splitting Applications: A First-Principles Investigation

Qiyun Deng [1,†], Lei Gao [1,2,*,†], Wuyi Gao [1], Jiali Hao [1], Chunhua Zeng [1] and Hua Wang [2,*]

[1] Institute of Physical and Engineering Science, Faculty of Science, Kunming University of Science and Technology, Kunming 650500, China; dengqiyun@stu.kust.edu.cn (Q.D.); haojiali@stu.kust.edu.cn (J.H.); chzeng83@kust.edu.cn (C.Z.)
[2] State Key Laboratory of Complex Nonferrous Metal Resources Clean Utilization, Kunming University of Science and Technology, Kunming 650093, China
* Correspondence: lgao@kust.edu.cn (L.G.); wanghua65@163.com (H.W.)
† These authors contributed equally to this work.

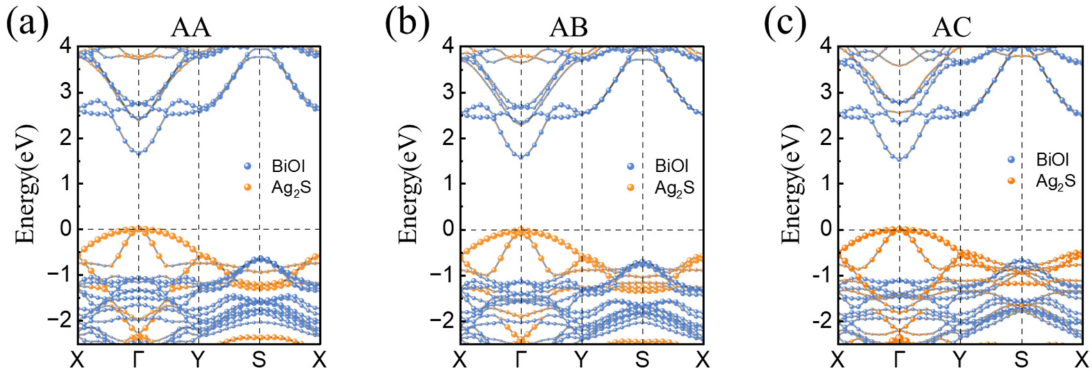

Figure S1. Projected band structures of Ag_2S/BiOI with (a) AA, (b) AB and (c) AC stackings, respectively.

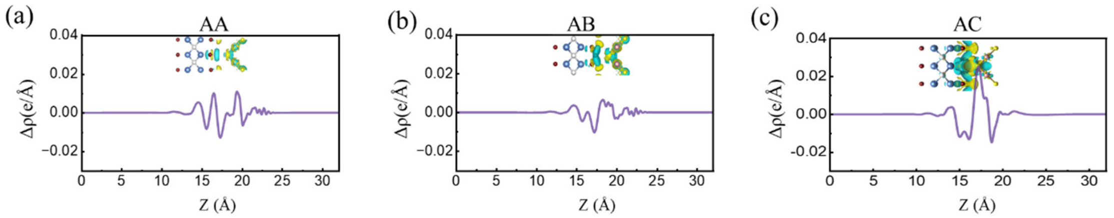

Figure S2. Charge density differences $\Delta\rho$ of Ag_2S/BiOI with (a) AA, (b) AB and (c) AC stackings, respectively.

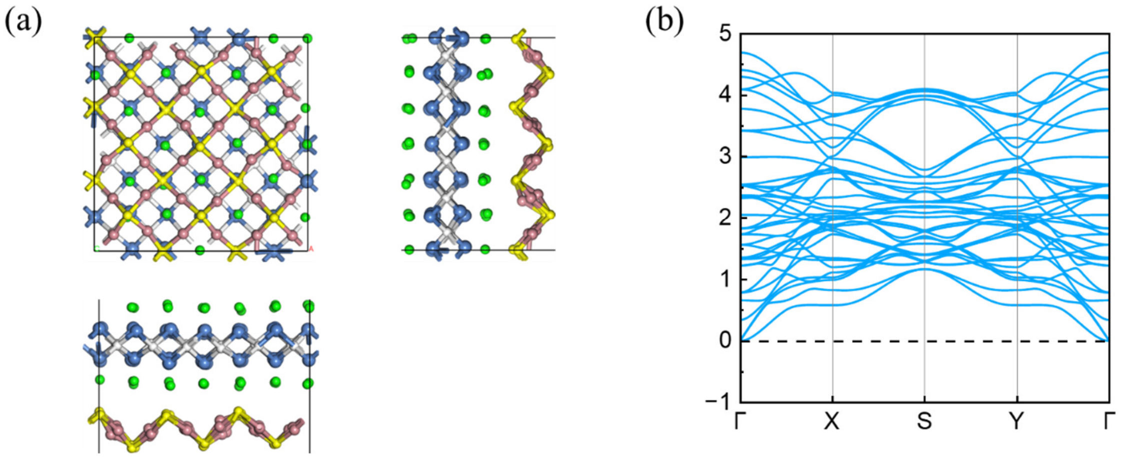

Figure S3. (a) Top and side views of the snapshots of Ag_2S/BiOI taken from ab initio molecular dynamic simulations carried out at 300 K for 2 ps. (b) Phonon dispersions of Ag_2S/BiOI.

Table S1. Comparison of Band Gaps between bulk and monolayer BiOX.

System	BiOCl	BiOBr	BiOI	Structure
Ref. 52	3.45	2.89	2.21	Bulk
This work	3.76	3.38	2.35	Monolayer

Table S2. The binding energy (E_b) of M_2X/BIOY heterojunctions.

E_b (eV)	Ag_2S	Ag_2Se	Au_2Se
BiOCl	-2.686	-2.592	-2.974
BiOBr	-2.996	-2.922	-3.318
BiOI	-3.304	-3.269	-3.646

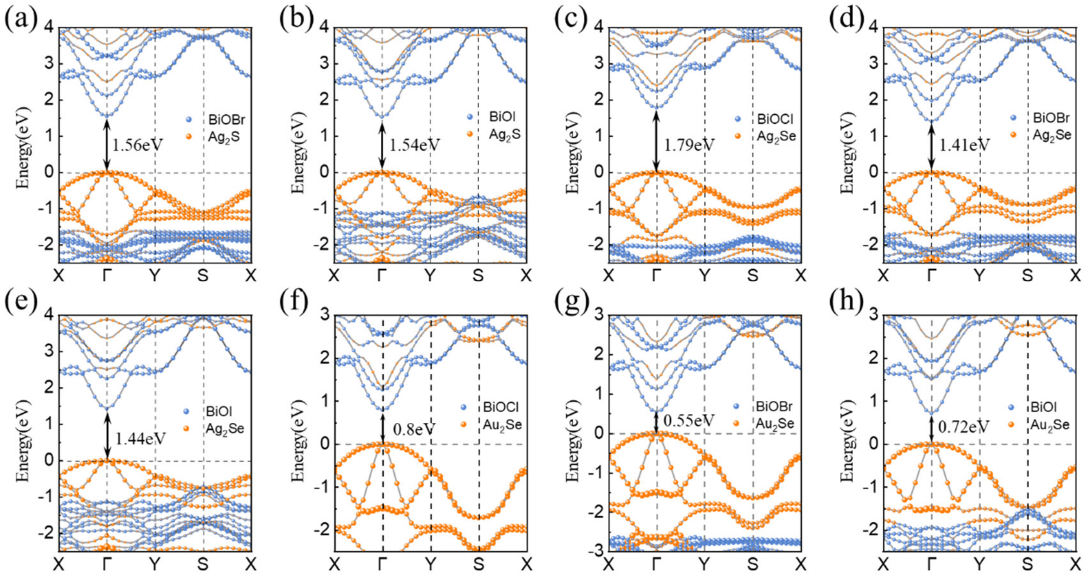

Figure S4. Projected band structures of M_2X/BiOY heterojunctions. The orange and blue colors represent the M_2X monolayer and the BiOY monolayer, respectively.

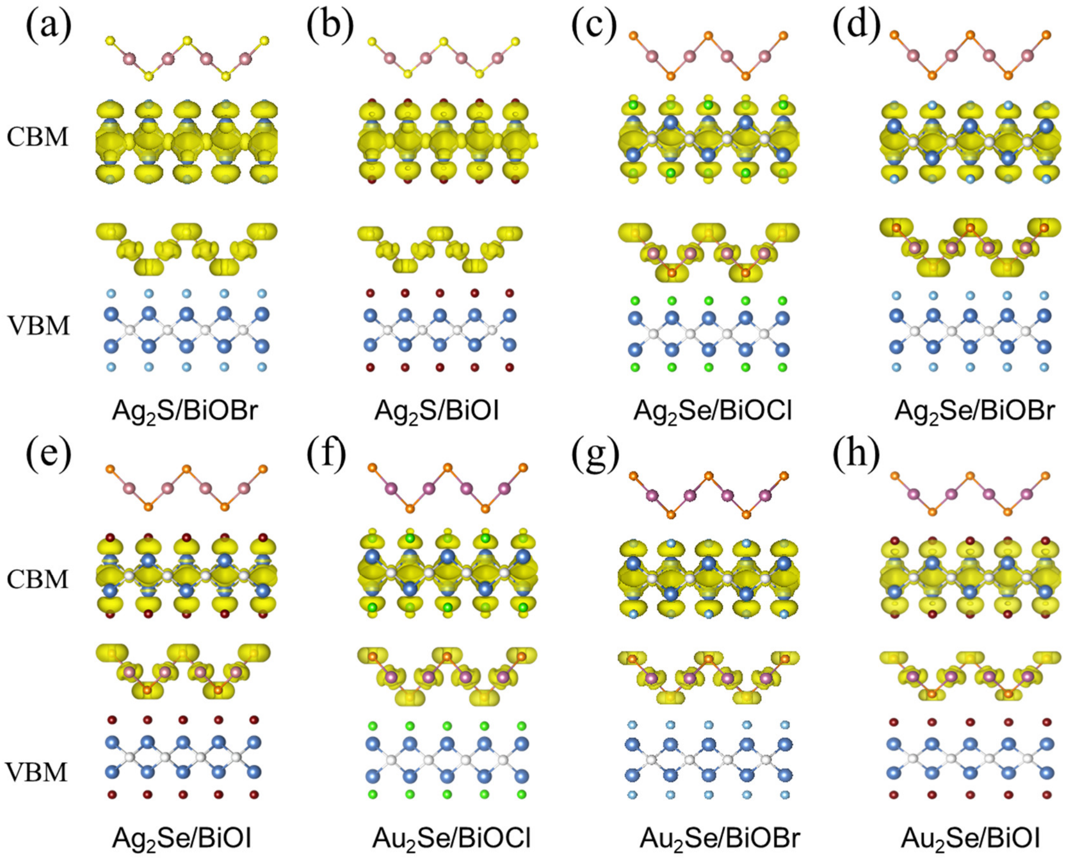

Figure S5. Partial charge densities of M_2X/BiOY heterojunctions.

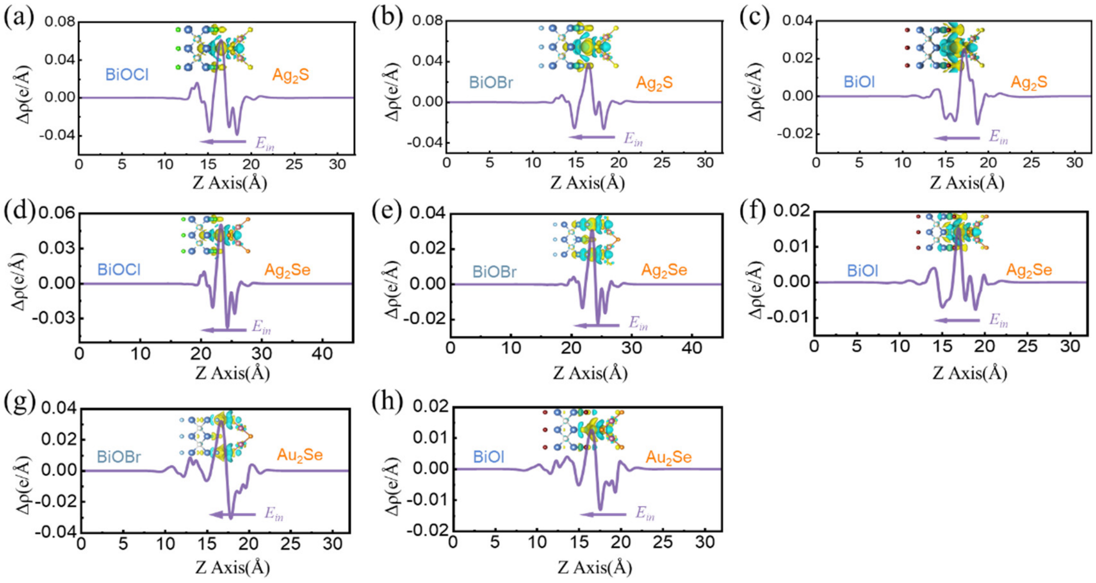

Figure S6. Planar-averaged charge density differences along the *z* direction of M_2X/BiOY heterojunctions.

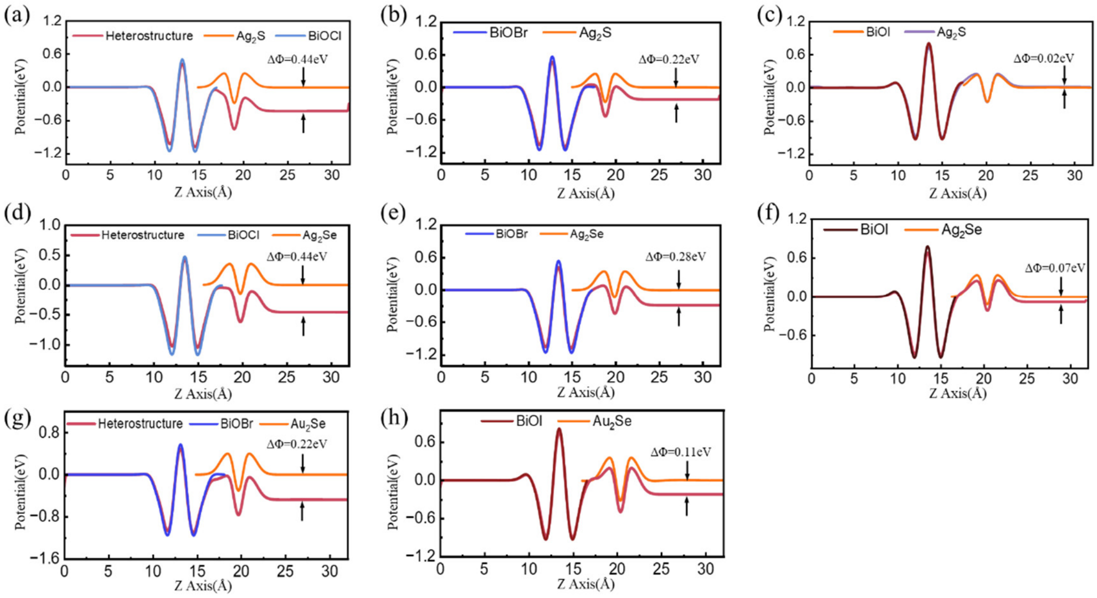

Figure S7. Plane-averaged Hartree difference potentials along the *z* direction of M_2X/BiOY heterojunctions.

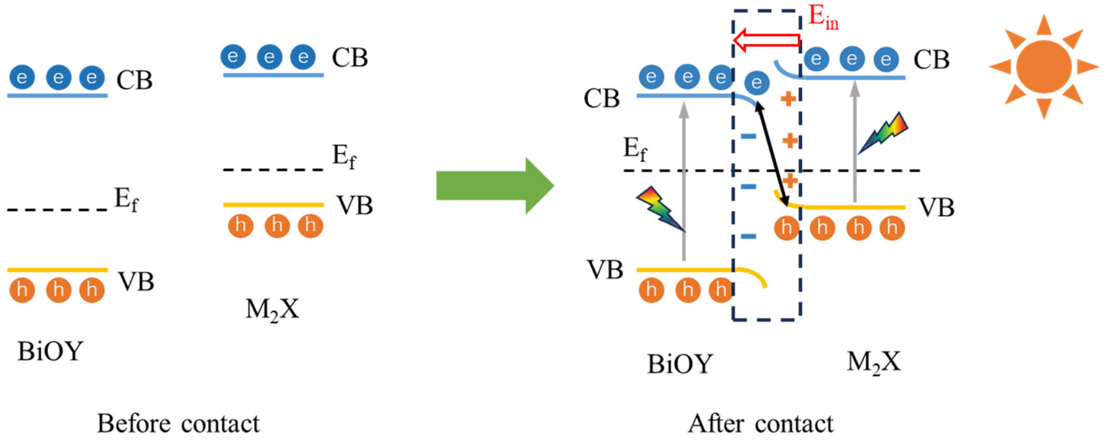

Figure S8. Fermi level change in M_2X/BiOY before contact and after contact.

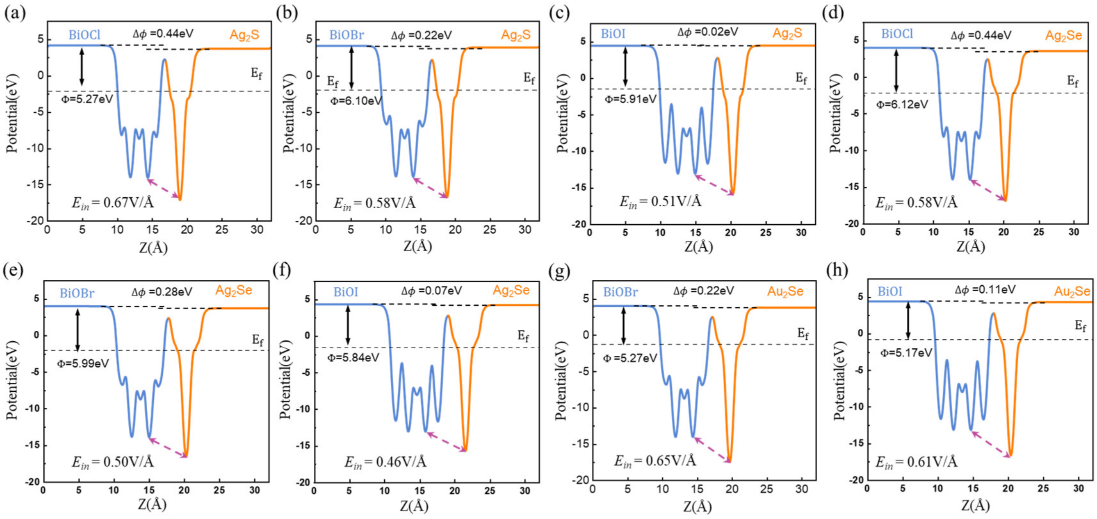

Figure S9. Plane-averaged electrostatic potential along the *z* direction of M_2X/BiOY heterojunctions. E_f represents the Fermi level and the black arrow indicates the difference between the Fermi level and the vacuum level. The vacuum energy level difference between the two sides of the heterojunction is represented by $\Delta\phi$.
